# Incidence and predictors of mortality in children with diabetic ketoacidosis in the comprehensive specialized referral hospitals of West Amhara Region, Northwest Ethiopia: a retrospective follow-up study

**DOI:** 10.3389/fcdhc.2023.1204133

**Published:** 2023-08-31

**Authors:** Rahel Asres Shimelash, Getaneh Mulualem Belay, Worknesh Aknaw, Aster Tadesse Shibabaw, Aderajew Agmas Adebabay, Gezahagn Demsu Gedefaw, Tadele Derbew Kassie, Alemu Birara Zemariam

**Affiliations:** ^1^Department of Pediatrics and Child Health Nursing, School of Nursing, College of Medicine and Health Sciences, Debre Markos University, Debre Markos, Ethiopia; ^2^Department of Pediatrics and Child Health Nursing, School of Nursing, College of Medicine and Health Sciences, University of Gondar, Gondar, Ethiopia; ^3^Department of Biomedical Science, School of Medicine, College of Health Sciences, Debre Markos University, Debre Markos, Ethiopia; ^4^Department of Neonatal Health Nursing, School of Nursing, College of Medicine and Health Sciences, University of Gondar Comprehensive Specialized Referral Hospital, Gondar, Ethiopia; ^5^Department of Public Health, College of Medicine and Health Sciences, Debre Markos University, Debre Markos, Ethiopia; ^6^Department of Pediatrics and Child Health Nursing, School of Nursing, College of Medicine and Health Sciences, Woldia University, Woldia, Ethiopia

**Keywords:** diabetic ketoacidosis, mortality, predictive factors, survival status, children

## Abstract

**Background:**

Diabetic ketoacidosis is one of the major life-threatening conditions associated with acute metabolic complications. It remains a major public health problem in developing countries such as Ethiopia.

**Objective:**

To assess the incidence and prediction of mortality in children with diabetic ketoacidosis in West Amhara Region Comprehensive Specialized Referral Hospitals, Northwest Ethiopia, in 2022.

**Methods:**

An institution-based retrospective follow-up study was conducted among 423 study participants with a confirmed diagnosis of diabetic ketoacidosis from 01/01/2017 to 31/12/2021. Data were entered, coded, cleaned, and checked using Epi-Data version 4.6 and exported to Stata version 14 for data analysis.

**Results:**

A total of 401 child records were included in the final analysis and were followed for 3781 days during the study period. The overall mortality of children with diabetic ketoacidosis was 10.6 per 1000 person-days observed (95% CI: 7.8-14.4) during the entire follow-up period. Hypoglycemia (AHR=4.6; 95% CI: 2.13-10.1), rural residence (AHR=2.9; 95% CI=1.01-8.11), age younger than five (AHR=4.4; 95% CI=1.4-13.7) or between five and 10 (AHR=3.1; 95% CI=1.1-8.8), and female gender (AHR=2.6; 95% CI=1.1-5.8) were significant predictors of mortality.

**Conclusions:**

The incidence rate of mortality in children with diabetic ketoacidosis was relatively high. Age, rural residence, female gender, and hypoglycemia were significantly predictive of mortality. Community education or mass campaigns about the signs and symptoms of diabetic ketoacidosis may reduce the mortality rate in children.

## Introduction

Diabetic ketoacidosis (DKA) is a common complication of type 1 diabetes mellitus in children (1). It was defined as hyperglycemia (blood glucose level greater than 13.9 mmol/L or greater than 250mg/dl and any of the following: venous PH less than 7.3 or bicarbonate less than 15 mmol/L). In addition, clinical signs and symptoms of volume depletion, Kussmaul breathing, cerebral edema, acetone or fruity breath odor, mental status alterations, vomiting, nausea, and infection are indicators of DKA (2, 3).

DKA is becoming increasingly common in children (4). It has a mortality rate of 24% (5). According to the World Health Organization report, DKA is the leading cause of death in the 21st century, with a mortality rate of 10% per patient each year in children (6, 7). DKA accounts for 20% of all pediatric deaths in the United States (8). According to the CDC’s United States Surveillance System (USSS), the mortality rate of children with DKA was 6.3% (9). In Africa, the mortality rate of children with DKA was reported to be nearly 7% (10).

The global economic impact of DKA on the healthcare system is significant. Because of the direct and indirect economic impact of DKA, there will be a total global loss of US$ 1.7 trillion from 2011 to 2030, with US$ 900 billion in high-income nations and US$ 800 billion in low- and middle-income countries, while the exact impact in Africa is unknown (11, 12). DKA also represents a crisis in terms of healthcare costs, with a single admission costing between US $4125 and US $11,196 (13).

Several studies found that different factors were significantly associated with the mortality rate of DKA in children, such as infections and discontinuation of insulin therapy (14). Also, another study showed that the main significant predictors of mortality in children with DKA are altered sensation, shock, delayed diagnosis, renal failure, and sepsis (15). Early recognition of complications, especially cerebral edema, could reduce the serious consequences in children (16). It is preferable to raise awareness among healthcare workers, teachers, children, and parents (15). According to a recent study, early detection, assistance, and supervision are important for better outcomes in children with DKA. Healthcare workers who treat children are recommended to follow the treatment guidelines properly (17).

The Ethiopian Federal Ministry Of Health (FMOH) has established a National Strategic Action Plan (NSAP) for four priority non-communicable diseases (NCDs), which include DKA. However, limited resources such as low socioeconomic status and fast urbanization have increased morbidity, disability, and mortality among children with DKA (18). DKA remains a major public health concern in underdeveloped countries and a leading cause of death in children in Sub-Saharan Africa, including Ethiopia (19). The fatality rate has not been investigated in Ethiopia, even in the study areas. Hence, the main aim of this study was to determine the incidence and predictors of mortality among children in DKA.

## Methods

### Study design and period

An institution-based retrospective follow-up study was conducted to review five-year medical records of children with DKA in the Comprehensive Specialized Referral Hospitals of West Amhara Region from 01/01/2017 to 31/12/2021. Data were retrieved from 01/05/2022 to 30/05/2022.

### Study setting and population

The University of Gondar, and the Felege-Hiwot, Tibebe-Ghion, Debre-Markos, and Debre-Tabor Comprehensive Specialized Referral Hospitals were the study settings. All children with DKA admitted at referral hospitals in West Amhara Region were the source population, and among them, those diagnosed with DKA from 01/01/2017 to 31/12/2021 were the study population.

### Eligibility criteria

All children with DKA less than 15 years of age with follow-up from 01/01/2017 to 31/12/2021 were included in this study. Children with incomplete baseline clinical data, lost medical records, and outcome variables not recorded in their medical records during the data collection period were excluded.

### Sample size determination and sampling procedures

The sample size was calculated differently based on incidence and prediction. Since the incidence of death among children with DKA is not yet studied in Ethiopia, the minimum required sample size was calculated using proportion (50%). Then the sample size was determined based on the single population proportion formula N= (Z∝/2)^2^ * p (1-p)/(d) ^2^Z ∝/2 where N= sample size, Z∝/2 corresponding Z-score of 95% CI (1.96), and d=margin of error (5%). Rearranging the given formula, N= (1.96)^2^ * 0.5 * 0.5/(0.05)^2 = ^384.16. Finally, assuming 10% incomplete data (38,416), the final sample size for incidence was calculated as 422,576 ≈423 .

For prediction, the sample size was determined by using the double population proportion formula and considering predictive variables of DKA mortality using the Epi-Info version 7 statistical package. It was calculated using a two-sided significant level ( ∝ ) of 5%, power (ZB) of 80%, and a 1:1 ratio of exposed to non-exposed at 95% CI. Two predictive variables, infection and missed insulin doses, were used to calculate the sample size.

In this study, P1= percent of death outcomes in exposed children, and P2= percent of death outcomes in non-exposed children, Z ∝/2 : is taking CI 95%, ZB is power, and r = 1:1. Based on the exposure status of the infection, p1 = 50.9% and p2 = 27.4%, respectively. After entering these data into Epi-Info, considering 10% incomplete data, the total sample size was =165. In terms of missed insulin doses, p1 = 48.5%, and p2 = 33.2%; by adding 10% incomplete data, the total sample size was 386.8 (20). Finally, a large sample size of N=423 was selected for this study.

### Sampling technique and procedures

Initially, the number of medical records of children with DKA was obtained from each hospital, and 670 children with DKA between 01/01/2017 and 31/12/2021 from five referral hospitals. Then, the sample size was proportionally allocated to each hospital. Based on this, 107 out of 170 from the University of Gondar Hospital, 103 out of 163 from Felege-Hiwot Hospital, 93 out of 147 from Debre Markos Hospital, 44 out of 70 from Tibebe-Ghion Hospital, and 76 out of 120 from Debre Tabor Hospital were taken popotionally. Finally, a computer-generated simple random sampling technique was employed to recruit the study participants. A total of 423 medical charts were reviewed, of which 22 records were discarded due to the exclusion criteria. In total, 401 medical records were used for the final analysis.

### Operational definitions

Event: Death of the child at the specific time of follow-up, as confirmed by the physician.

Diabetic ketoacidosis: Defined as hyperglycemia (blood glucose > 250 mg/dl or 13.9 mmol/L and any of the following: venous PH<7.3 or bicarbonate< 15 mmol/L) (21).

Baseline clinical data: An initial measurement of a condition taken early and used for comparison over time to look for changes (22).

### Data collection tools and procedures

The data extraction tool was adapted from reviewed literature sources (23, 24). The questionnaire was given to different experts working in different specialties, like neonatology and pediatric nursing, to check its internal validity. Data were collected from patient charts. The charts were accessed based on their medical record number. Then, all DKA medical record numbers (MRN) were selected. Following the lottery method, simple random sampling was used. Three BSc nurses were recruited for supervision, and one BSc nurse was recruited for data collection for each hospital. Training was given for one day to data collectors and supervisors on data collection tools and procedures.

### Data processing and analysis

Data were entered, coded, cleaned, and checked using Epi-Data version 4.6, then checked for completeness and consistency and exported to Stata version 14 for data analysis. Descriptive measures were used to characterize the data. Missing data were handled by multiple imputations. Kaplan-Meier and log-rank tests were used to estimate and compare survival times. The incidence rate of DKA mortality during the follow-up period was calculated and presented per 1000 person-day observations. The overall cumulative survival rate from the total number of child person-days observed during the follow-up period was 3781 person-days, with a median time of 31 days. Children were followed for a minimum of 1 and a maximum of 33 days in the inpatient department. The assumption of the proportional hazards model was tested by using the Schoenfeld residuals test together with the graphical test. In addition, the overall goodness of the model fit was checked via the Cox-Snell residuals. So, it can be concluded that the final model fits the data well. To investigate the prediction of mortality in children with DKA, the Cox proportional-hazards model was employed, and bivariable Cox regression analysis was performed: variables with *P*< 0.25 levels were eligible for multivariable analysis. The AHR, 95% CI, and *P*-value were used to assess the strength of the association and statistical significance. All variables with a *P*-value< 0.05 in the multivariable analysis were considered statistically significant in predicting DKA mortality.

## Results

### Socio-demographic characteristics of the participants

A total of 401 children were enrolled in the study. The completion rate was 95%. Nearly half of the children, 215 (53.62%), were female. The mean age of the children with DKA was 8.5 (± 2) years. Nearly two-thirds of the children, 249 (62.09%) had primary and higher education, and 252 (62.84%) were from rural areas (**Table 1**).

**Table 1:** Socio-demographic characteristics for prediction of mortality in children with diabetic ketoacidosis in the Comprehensive Specialized Referral Hospitals of West Amhara Region, Northwest Ethiopia, 2022 (N=401).

Variables	Category	Frequency	Percent (%)
Gender	Male	186	46.38
	Female	215	53.62
Age	< 5 years old	91	22.69
	5-10 years old	152	37.91
	11-14 years old	158	39.4
Religion	Orthodox	354	88.28
	Muslim	26	6.48
	Other	22	5.24
Residence	Rural	252	62.84
	Urban	149	37.16
Child’s caregiver	Mother	224	55.86
	Father	151	37.66
	Other	26	6.48
Child’s education status	Not started	66	16.46
	KG	86	21.45
	Primary and above	249	62.09

### Treatment factors for prediction of mortality in children with DKA

Nearly half of the children, 214 (53.37%), had good medication adherence, while 229 (57.11%) had improper medication storage, and almost half of the children, 198 (49.38%), had discontinued medication (**Table 2**).

**Table 2:** Clinical characteristics and baseline data for predicting mortality in children with diabetic ketoacidosis in the Comprehensive Specialized Referral Hospitals of West Amhara Region, Northwest Ethiopia, 2022 (N=401).

Variable	Category	Frequency	Percent (%)
Child’s family history of DM	Yes	280	69.83
	No	121	30.17
Child diagnosed with DM	New	155	38.65
	Known	246	61.35
Recurrence of DKA	One time	154	38.4
	Two times	85	21.2
	Three times	128	31.92
	Four or more	34	8.48
Severity of DKA	Mild	84	20.95
	Moderate	156	38.5
	Severe	161	40.15
Level of consciousness on admission	Mild	219	54.61
	Moderate	158	39.4
	Severe	24	5.99
Vomiting on admission	Yes	263	65.59
	No	138	34.41
Infection	Yes	248	61.85
	No	153	38.15
Co-morbidity	Yes	136	33.92
	No	265	66.08
Complications	Yes	71	17.71
	No	330	82.29
Median (interquartile range)			
Fasting blood glucose (mg/dl)	365 (301, 428)		
Random blood glucose (mg/dl)	501 (450, 584)		
Ketones	3 (2, 3)		
Urine glucose	3 (2, 3)		

### Assessing the proportional hazard assumption

The Schoenfield residuals were used as the basis for the global test of the proportional hazard assumption, which had a P-value of 0.67. When examining several multivariable Cox regression analyses, the minimum and maximum P-values were 0.06 and 0.92, respectively.

### Incidence of mortality and overall failure function

**Figure 1:**
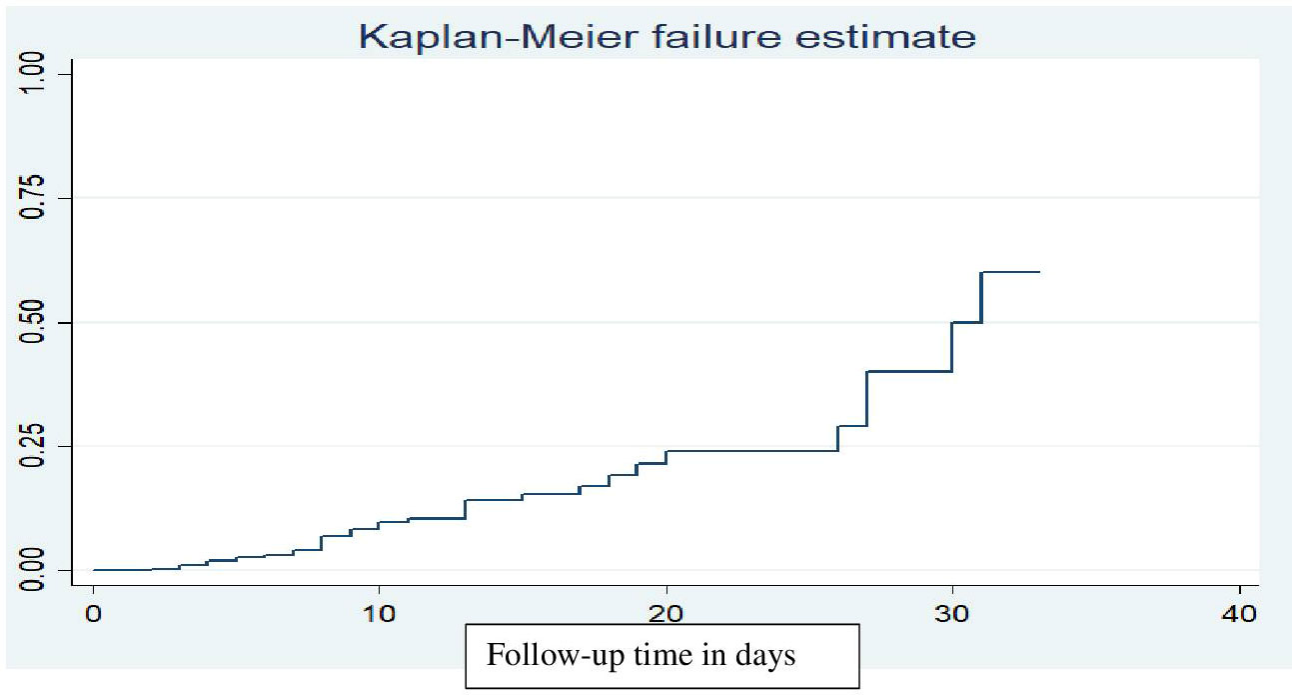
Kaplan-Meier failure function estimate of mortality in children with diabetic ketoacidosis in the Comprehensive Specialized Referral Hospitals of West Amhara Region, Northwest Ethiopia, 2022 (N=401).

Out of 401 children, 40 (10%) died during the follow-up period. The overall cumulative survival rate from the total days of observation of the children during the follow-up period was 3781 person-days, with a median time of 31 days. Children were followed for a minimum of 1 and a maximum of 33 days in the inpatient department. Throughout the entire follow-up period, the overall mortality rate for children with DKA was 10.6 per 1000 person-days of observation (95% CI: 7.8-14.4). The overall Kaplan-Meier failure function indicated that the probability of death increased during the follow-up period. The cumulative odds of death at 10, 20, and 30 days were 0.08, 0.16, and 0.36, respectively (**Figure 1**).

### Fitness of the model

**Figure 2:**
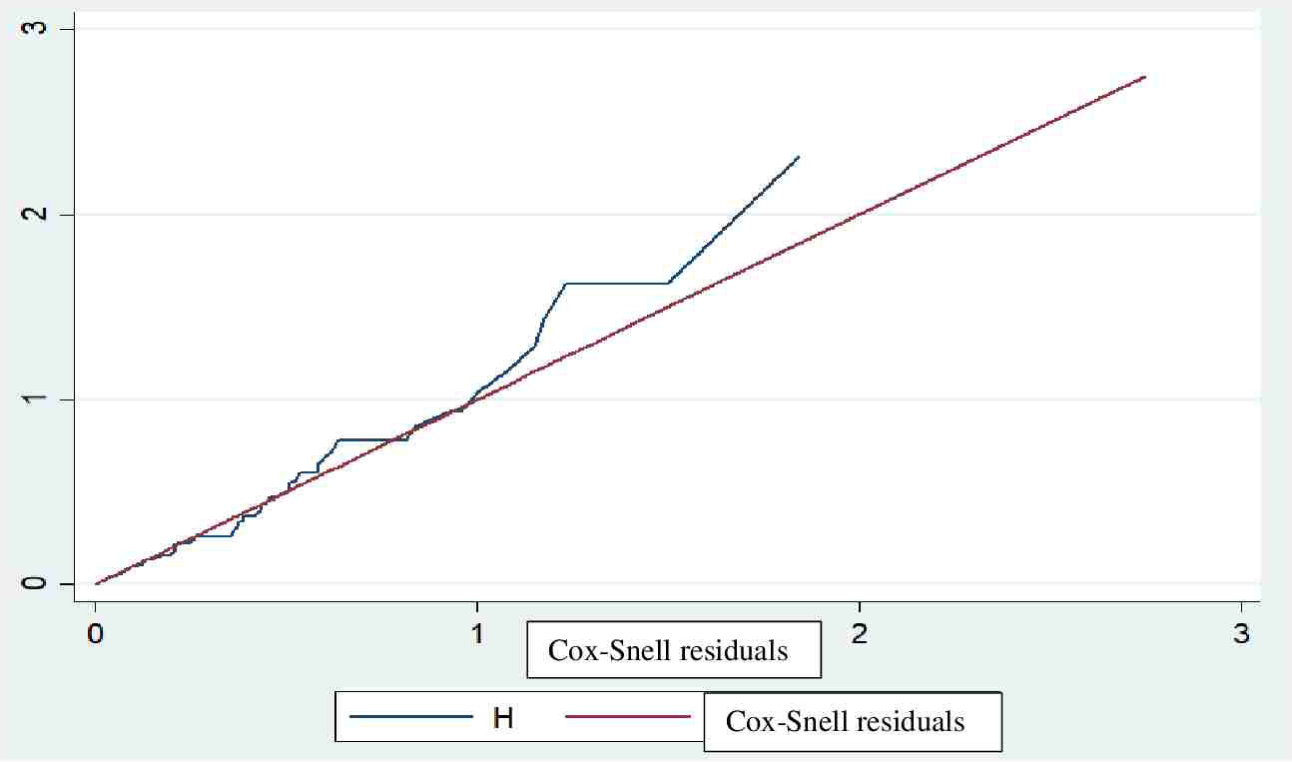
Fitness of model confirmed with Cox-Snell residuals for mortality in children with diabetic ketoacidosis in the Comprehensive Specialized Referral Hospitals of West Amhara Region, Northwest Ethiopia, 2022 (N=401).

The fitness of the model was checked graphically using the Cox–Snell residuals and the Nelson-Aalen cumulative hazard graph, as the line follows 45 degrees (**Figure 2**).

### Predictors of mortality in children with DKA

In bivariate Cox regression, the severity of diabetic ketoacidosis, Glasgow Coma Scale, vomiting, complications, place of residence, medication discontinuation/skipping, infection, age, gender, co-morbidity, education status, drug adherence, and recurrent diabetic ketoacidosis were significantly predictive of mortality in DKA children at a P-value lower than or equal to 0.25. On the other hand, multivariate Cox regression analyses like hypoglycemia, living in a rural area, age, and female gender were significantly predictive of mortality in children with diabetic ketoacidosis at a P-value lower than 0.05 (**Table 3**).

**Table 3:** Bi-variable and multi-variable Cox regression analyses for predicting mortality in children with diabetic ketoacidosis in the Comprehensive Specialized Referral Hospitals of West Amhara Region, Northwest Ethiopia, 2022 (N=401).

Variables	Category	Survival status		CHR (95% CI)	AHR (95% CI)	P-value
		Event	Censored			
Age	<5	19	72	0.77 (0.58, 1.02)	4.38 (1.41, 13.7)	0.011*
	5-10	15	137	0.93 (0.74,1.17)	3.06 (1.06, 8.8)	0.038*
	11-14	6	152	1	1	
Gender	Male	10	176	1	1	
	Female	30	185	0.79 (0.65, 0.98)	2.5 (1.14, 5.79)	0.023*
Residence	Urban	5	144	1	1	
	Rural	35	217	0.79 (0.64, 0.97)	2.86 (1.01,8.11)	0.049*
Child’s education status	Not started	16	49	0.71 (0.52, 0.96)	2.01 (0.76,5.35)	0.162
	KG	10	76	0.84 (0.65, 1.09)	0.95 (0.38,2.38)	0.906
	Primary	14	236	1	1	
Infection	Yes	35	213	0.73 (0.59, 0.90)	1.2 (0.397,3.59)	0.753
	No	5	148	1	1	
Co-morbidity	Yes	24	112	0.59 (0.48, 0.75)	0.96 (0.45,2.05)	0.914
	No	16	249	1	1	
Hypoglycemia	Yes	24	47	0.49 (0.36, 0.68)	4.63 (2.13,10.06)	0.001*
	No	16	314	1	1	
Medication adherence	Yes	12	202	1.52 (1.21, 1.82)	0.74 (0.32,1.71)	0.481
	No	28	159	1	1	
Recurrent DKA diagnosed	One time	9	145	1	1	
	Two times	10	75	0.99 (0.75, 1.31)	1.46 (0.55, 3.86)	0.45
	Three times	11	117	0.93 (0.73, 1.18)	1.3 (0.5, 3.31)	0.59
	Four or more	10	24	0.47 (0.30,0.74)	0.86 (0.19, 3.85)	0.85
Severity of DKA	Mild	5	79	1	1	
	Moderate	10	146	0.75 (0.57, 0,99)	0.56 (0.18, 1.83)	0.330
	Severe	25	136	0.66 (0.51, 0.88)	1.04 (0.32, 3.49)	0.953
Glasgow Coma Scale	Mild	12	207	1	1	
	Moderate	19	139	0.96 (0,77, 1.21)	1.2 (0.45, 2.83)	0.790
	Severe	9	15	0.33 (0.19, 0.57)	1.06 (0.91, 2.32)	0.340
Vomiting	Yes	33	230	1.01 (0.81,1.25)	1.81 (0.65, 5.02)	0,254
	No	7	131	1	1	

* indicates the significant variables which is P-value less than 0.05.

The hazard of death in children with hypoglycemia was 4.6 times higher than in those without (AHR=4.6; 95% CI: 2.13-10.1). Rural residence increased the hazard of death by a factor of 2.9 compared to urban residence (AHR=2.9; 95% CI=1.01-8.11). In children younger than five years, the hazard of death increased 4.4-fold (AHR=4.4; 95% CI=1.4-13.7), and the hazard of death increased by a factor of 3.1 times in children aged five to 10 years compared to children aged 11 to 14 years (AHR=3.1; 95% CI=1.1-8.8). The hazard of death was 2.6 times higher in female children compared to male children (AHR=2.6; 95% CI=1.1-5.8).

## Discussion

In this study, the mortality rate was 10%. The overall mortality of children with diabetic ketoacidosis was 10.6 per 1000 child days observed (95% CI: 7.8-14.4) during the entire follow-up period. This finding was in line with studies conducted in Bangladesh, where the all-time mortality was 13.4/1000 (25), in Korea (11.8/1000) (26), and in South India (11/1000) (15). On the other hand, the results of the current study were relatively higher compared to other research conducted in the USA, where the all-time mortality was 0.21/1000 (27), Canada (0.18/1000) (28), UK (0.31/1000) (29), Iran (1.7/1000) (30), Kenya (6.9/1000) (17), and Pakistan (3.4/1000) (31). This discrepancy may be due to lifestyle differences between these populations and the population under study. Lifestyle modifications such as a healthy diet and regular physical activity might reduce the mortality rate of children with DKA in these countries. A low economic societal status, lack of access to health care facilities, and limited resources in the current study country may increase the mortality of children with DKA (32). Conversely, this finding was lower than a study conducted in Turkey, where the mortality of children with diabetic ketoacidosis was 24% (33). The possible reason for this may be due to the fact that the previous study was a prospective cohort and proper event registration may have occurred, but the current study was a retrospective cohort, and children who died due to DKA may have been overlooked.

Rural residence increased the hazard of death by a factor of 2.9. This finding was supported by a study conducted in Iraq (34). Rural residents were more susceptible to mortality compared to urban residents. Since most rural communities lack infrastructure such as health facilities and transportation, subjects may not be able to reach a health center as quickly as possible during their illness. Additionally, those living in remote areas with low socioeconomic levels may find it challenging to pay for transportation, medications, and medical monitoring equipment (35, 36). The other possible reason may be that people living in a rural setting may have lower health-seeking behaviors as compared with those living in urban areas (37).

Hypoglycemia was a significant predictor of mortality in children with DKA. Hypoglycemia increased the risk of death by a factor of 4.6. This finding was supported by a study conducted in India (38). The reason may be that if hypoglycemia is not treated in time, the condition may quickly deteriorate. Prolonged hypoglycemia leads to encephalopathy, brain damage, cardio-respiratory arrest, severe arrhythmia, and ischemic cardiovascular attack, along with loss of consciousness, coma, and death. (39).

Age was a significant predictor of mortality in children with DKA. Ages under five and between five and 10 increased the risk of death by 4.4 and 3.1 times, respectively, compared to ages between 11 to 14. This finding was supported by studies conducted in the whole of the UK (40) and in the city of Birmingham (41). The child’s age brings its own set of challenges when it comes to diabetes management. Because smaller children are more dependent on their caregivers from infancy through preschool, which results in non-adherence to medication and diet, difficulties in understanding clinical signs and symptoms, refusal to modify their lifestyle, and failure to convey their hypoglycemia and hyperglycemia symptoms, this could cause blood glucose level fluctuations and death (42–44).

Gender was one of the independent significant predictors of mortality in children with DKA. Female children had a 2.6-fold increased risk of death compared to male children. This finding was supported by a study conducted in Germany (45). One possible reason may be due to puberty-associated hormonal changes, such as a change in estrogen levels. Also, female children may have body image issues, particularly in adolescence, which results in skipping meals and insulin injections for weight loss (46). Furthermore, this may be due to lower sensitivity to insulin in female subjects than in male subjects. Female children have higher levels of glycosylated hemoglobin A1C (HgA1C), cholesterol, low-density lipoprotein (LDL), and triglycerides during the pre-pubertal and pubertal stages, leading to poorer glycemic control in female children (7, 47, 48).

### Study limitations

Since our data were collected from secondary sources, the main limitation of this research consists of incomplete medical records. This may affect the outcomes of the study. Some important maternal and paternal predictive variables, such as educational level, and monthly income, in addition to baseline clinical data like PH, bicarbonate, and HgA1C were not reported in the medical records. Furthermore, since the data were collected in a hospital setting, child mortality in DKA patients may be underestimated. The child may die at home, and as a result, the mortality rate may be underreported.

## Conclusions and recommendations

The current study showed that the overall incidence of mortality in children with DKA was relatively high as compared to the reports on diabetes from the Ministry of Health in the Federal Democratic Republic of Ethiopia. Hypoglycemia, age below 10 years, rural residence, and female gender were significant predictors of mortality in children with DKA in the West Amhara Region. The key to achieving good outcomes and preventing mortality is the prevention of DKA through early detection, support, supervision, and proper blood glucose monitoring. Community education or mass campaigns on the signs and symptoms of DKA can reduce the death rate in children. Therefore, it is important that healthcare workers treating children with DKA follow guidelines. Finally, further prospective longitudinal studies would be recommended by incorporating important predictors of DKA mortality, such as baseline laboratory investigations of PH levels, bicarbonate, and HgA1C, and including other predictors like socio-economic status in addition to paternal or maternal educational levels.

## Patient and public involvement

Patients were not formally involved in the development of this specific study design.

## Data availability statement

The original contributions presented in the study are included in the article/supplementary material, further inquiries can be directed to the corresponding author/s.

## Ethics statement

Ethical approval was obtained from the School of Nursing Ethical Review Committee on behalf of the Institutional Research Ethical Committee of the University of Gondar, College of Medicine and Health Sciences, with reference number (248/2022) on 14/5/2022. Written informed consent from the participants' legal guardian/next of kin was not required to participate in this study in accordance with the national legislation and the institutional requirements.

## Author contributions

RS: design, methodology, software, analysis, resources, data curation, draft of the manuscript, editing, visualization, and supervision. GB and WA: collaborated in visualization, methodology, software, data curation, analysis, review of the manuscript draft, and supervision. AZ, AS, AA, GG, and TK: methodology, data curation, analysis, review of the manuscript draft, and supervision. All authors contributed to the article and approved the submitted version.
